# The Effect of Galvanic Vestibular Stimulation on Visuospatial Cognition in an Incomplete Bilateral Vestibular Deafferentation Mouse Model

**DOI:** 10.3389/fneur.2022.857736

**Published:** 2022-03-18

**Authors:** Thanh Tin Nguyen, Gi-Sung Nam, Gyu Cheol Han, Chuyen Le, Sun-Young Oh

**Affiliations:** ^1^Jeonbuk National University College of Medicine, Jeonju, South Korea; ^2^Department of Neurology, Jeonbuk National University Hospital and School of Medicine, Jeonju, South Korea; ^3^Department of Pharmacology, Hue University of Medicine and Pharmacy, Hue University, Hue, Vietnam; ^4^Department of Otorhinolaryngology-Head and Neck Surgery, Chosun University College of Medicine, Gwangju, South Korea; ^5^Department of Otolaryngology-Head and Neck Surgery, Gachon University of Medicine and Science, Graduate School of Medicine, Incheon, South Korea; ^6^Department of General-Endocrinology and Internal Medicine, Hue University Hospital, Hue, Vietnam; ^7^Research Institute of Clinical Medicine of Jeonbuk National University-Biomedical Research Institute of Jeonbuk National University Hospital, Jeonju, South Korea

**Keywords:** vestibular, labyrinthectomy, galvanic vestibular stimulation, functional recovery, spatial navigation, higher vestibular cognition

## Abstract

**Objectives:**

To evaluate the efficacy of galvanic vestibular stimulation (GVS) for recovering from the locomotor and spatial memory deficits of a murine bilateral vestibular deafferentation (BVD) model.

**Methods:**

Male C57BL/6 mice (*n* = 36) were assigned to three groups: bilateral labyrinthectomy with (BVD_GVS group) and without (BVD_non-GVS group) the GVS intervention, and a control group with the sham operation. We used the open field and Y maze, and Morris water maze (MWM) tests to assess locomotor and visuospatial cognitive performance before (baseline) and 3, 7, and 14 days after surgical bilateral labyrinthectomy. For the GVS group, a sinusoidal current at the frequency at 1 Hz and amplitude 0.1 mA was delivered for 30 min daily from the postoperative day (POD) 0 to 4 *via* electrodes inserted subcutaneously close to both the bony labyrinths.

**Results:**

Short-term spatial memory was significantly impaired in bilaterally labyrinthectomized mice (BVD_non-GVS group), as reflected by decreased spontaneous alternation performance in the place recognition test and time spent in the novel arm and increased same arm return in the Y-maze test, compared with the control. Long-term spatial memory was also impaired, as indicated by a longer escape latency in the hidden platform trial and a lower percentage of time spent in the target quadrant in the probe trial of the MWM. GVS application significantly accelerated the recovery of locomotion and short-term and long-term spatial memory deficits in the BVD mice.

**Conclusions:**

Our data demonstrate that locomotion, short-term, and long-term (at least 2 weeks) spatial memory were impaired in BVD mice. The early administration of sinusoidal GVS accelerated the recovery of those locomotion and spatial memory deficiencies. GVS could be applied to patients with BVD to improve their locomotion and vestibular cognitive functioning.

## Introduction

Given that anatomical end organs are extremely sensitive to physical acceleration, the vestibular system is needed to coordinate motion and balance (1, 2). To adequately accomplish that complex task, the vestibular system possesses an incredibly delicate sensory synergy and is linked to motor organs through reflexes such as the vestibulo-ocular reflex (VOR), vestibulospinal reflex (VSR), and vestibulocollic reflex (3). In addition, the vestibular system plays a highly significant role in cognitive functions, particularly visuospatial memory (2, 4, 5). Recently, imaging and histologic evidence have proposed pathways, including thalamocortical, theta-generating, cerebellocortical, head direction pathways (2, 6, 7), from the peripheral vestibular organs to the hippocampus, which is involved in the multiple learning and memory processes (8–10). Once it arrives in the brain, the vestibular information derived from peripheral organs swiftly becomes convergent, multisensory-integrated (1, 2, 11, 12). Accordingly, the loss of vestibular inputs causes not only vertigo, dizziness, visual instability, and disturbed balance (because of impairment of the vestibulo-thalamo-cortical pathways, VOR, and VSR), but also deficits in spatial navigation and memory tasks, body representation, and bodily self-consciousness (through dysfunctions in the vestibular cortex and hippocampus) (13). Although unilateral vestibular deafferentation (UVD) might cause some degree of visuospatial memory deficits (14–16), bilateral vestibular deafferentation (BVD) can create prominent spatial cognitive deficiencies (17–19) that can last for up to 10 years (20). Furthermore, bilateral hippocampal atrophy was documented following BVD in the clinical investigations (20, 21).

Vestibular deafferentation induces several functional and structural changes in the brain (21), including vestibular compensation, which encompasses restoration, habituation, and adaptation (21). Vestibular compensation takes place in various brain regions, including the vestibular nuclei, spinal cord, cerebellum, and cortices with different biochemical substrates (22), in an attempt to partially ameliorate the vestibular dysfunctions. Clinical prognosis in BVD is usually poor compared with the unilateral vestibular loss (21) because in BVD, peripheral recovery is incomplete, and the central mechanisms are confined to sensory substitution, including lowering the thresholds of other sensory (auditory, visual, proprioceptive, and tactile) processing and boosting reciprocal intersensory interactions. Therefore, a substantial proportion of patients with bilateral vestibulopathy do not recover significantly and live with prolonged spatial memory problems (23). Complete BVD animal models that use vestibular neurectomy, chemical labyrinthectomy, or complete removal of both the otolith organ and the semicircular canals (SCCs) (17–19, 24, 25) have produced permanent impairments in the spatial cognition (17, 18, 25). In this study, we used an incomplete BVD model by causing confined bilateral damage to only the posterior SCCs. Given that a restricted injury level is accompanied by a better possibility of recovery, our incomplete BVD model appears to be a promising candidate for evaluating new rehabilitation strategies for bilateral vestibular-related deficits.

Over the past 100 years, galvanic vestibular stimulation (GVS) has been extensively applied in the pathological situations to examine the role of vestibular signals in visual stability, postural balance, locomotor control, and spatial cognition (26–28). By modulating the firing rate of vestibular afferents of both vestibular nerves and hair cells, GVS has been demonstrated to ameliorate several vestibular-related functional deficits, not only visual stability, motor coordination, and posture but also cognitive and memory impairments, particularly in UVD (14, 15, 29, 30). Accordingly, 5 sessions of direct current, bilateral, bipolar GVS improved UVD-induced short- and long-term spatial memory deficits when the cathode (excitatory) was placed on the lesion side (14, 15). We designed this study to evaluate the efficacy of GVS for recovery from the spatial memory and navigation deficits caused by the vestibular deafferentation in a murine BVD model.

## Methods

### Animals

A total of thirty-six 9-week-old male C57BL/6 mice (Animal Technology, Koatech, Kyonggi-Do, Korea), weighing 20–25 g, were randomly allocated into 3 groups: bilateral labyrinthectomy (BL) with GVS intervention (BVD_GVS group, *n* = 12), BL without GVS intervention (BVD_non-GVS group, *n* = 12), and the control group (*n* = 12). The mice were housed separately and kept in the laboratory conditions with *ad libitum* feeding. All the efforts were made to reduce the number of mice used and to minimize potential suffering.

Surgical labyrinthectomy was chosen because of its relative simplicity, reliability, instantly symptom-induced, and faster-recovered ability in comparison to vestibular neurectomy or chemical labyrinthectomy. The surgical BL procedure was done in the BVD_GVS and BVD_non-GVS groups, which were described in our published studies (14, 15, 30). Through a small hole made in the posterior SCC with a diamond otologic drill, the perilymph fluid was aspirated for 3 min, and then the hole was filled with collagen to prevent further leakage (14, 15, 30). Using this surgical approach, we created a minor lesion (incomplete) in the vestibular apparatus while retaining the intact auditory organ. Meanwhile, sham surgery without labyrinthectomy was applied for mice in the control group. All the surgical and GVS application-prepared procedures were conducted under inhaled anesthesia by isoflurane gas (Ifran, O_2_ 5 L/min, 2.0, Hana Pharm Co. Ltd., Kyonggi-Do, Korea).

The animal procedures used in this study were consistent with the Assessment and Accreditation of Laboratory Animal Care International and were reviewed and approved by the Animal Care Committee of the Gachon University of Medicine and Science (IRB MRI2019-0008).

### Study Design

**Figure 1:**
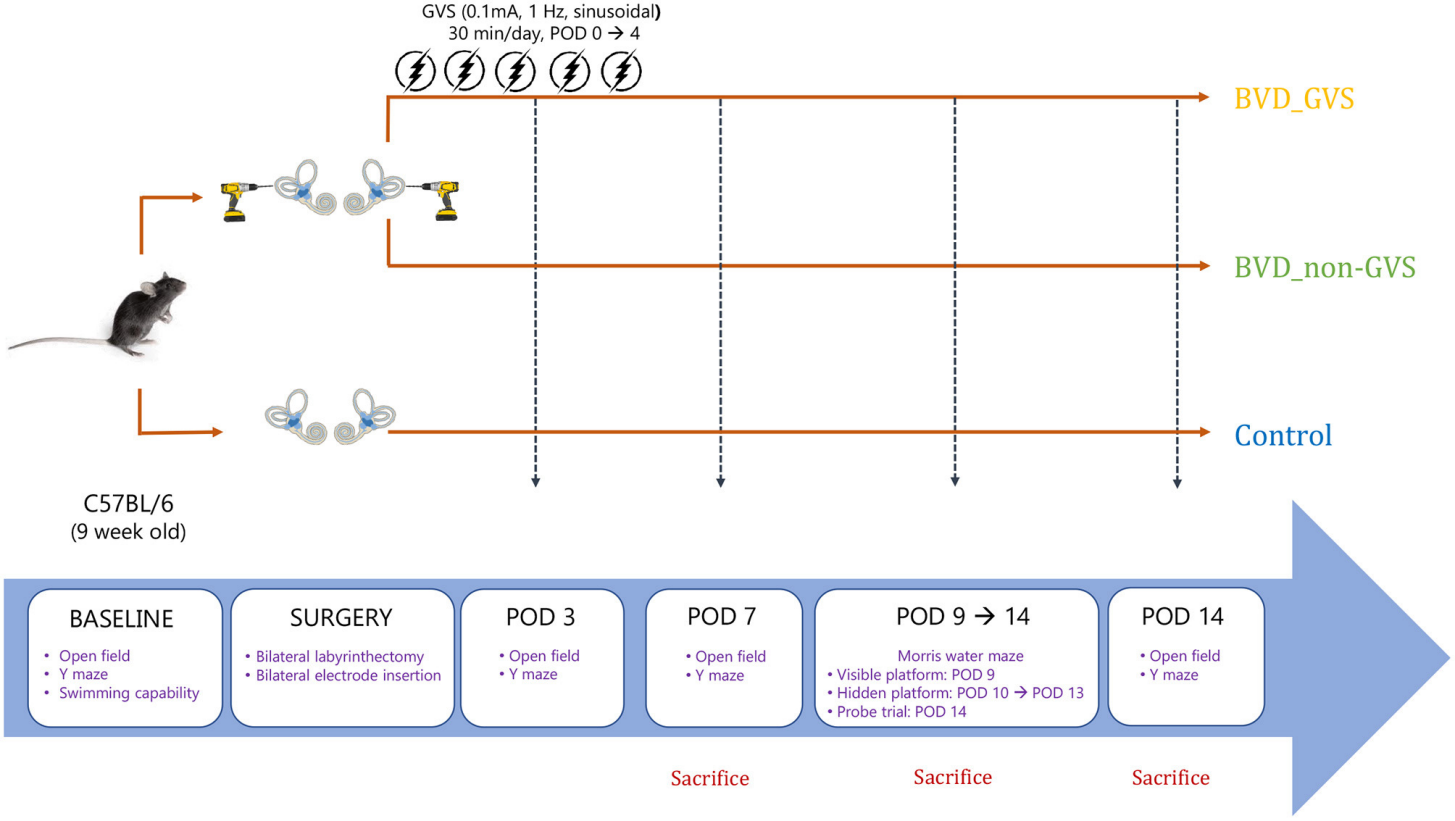
A schematic representation of the experimental design and schedules for GVS application. BVD, bilateral vestibular deafferentation; GVS, galvanic vestibular stimulation; POD, postoperative day.

To acquire baseline levels for motor and swimming capacity, we evaluated swimming ability and the open field (OF) and Y maze tests before conducting BL. Only mice that showed freely move and swimming were assigned to the three experimental groups. Following the surgery, the OF and Y maze behavioral tests were checked on postoperative days (PODs) 3, 7, and 14 to determine locomotor activities and spatial cognition. Meanwhile, the Morris water maze (MWM) training sessions were conducted for 5 consecutive days from POD 9 to 13, and the probe trial was performed on POD 14 to measure the long-term spatial memory (Figure 1). All the behavioral assessments were performed at the time between 11:00–15:00 to reduce the time-of-day impacts on the locomotor and exploratory activities.

The GVS application was implemented with the same paradigm of the electrode implantation and technical system described in our earlier works (14, 15, 30). As we have highlighted the beneficial effects of subthreshold GVS in our earlier studies with UVD model, we applied a subthreshold current in this study (14, 15, 30). Based on the analog-model pilot experiment, the GVS threshold was determined using oculomotor threshold or GVS-induced nystagmus monitoring which is a feasible and widespread applied approach in animals. As a result, a subthreshold, bipolar, sinusoidal GVS current of 0.1 mA and 1 Hz was generated by a computer-controlled stimulator (A-M Systems Model 2100 Isolated Stimulator, A-M Systems Inc., United States, and CED micro1401-4 Cambridge Electronic Design Ltd, United Kingdom) and delivered during a 30-min period daily, consecutively for 5 days. The restraint procedure during GVS application with no current was likewise applied for mice in the control and BVD_non-GVS groups.

#### Behavioral Observations

Assessments of the air-righting reflex and contact righting were used to behaviorally evaluate vestibular function following BL. In the air-righting reflex test, we held the mice in a supine position and dropped (roughly 40 cm) onto a soft surface. Meanwhile, in the contact-righting test, we laid the mice supine on a horizontal surface and another horizontal surface was placed in contact with their feet. Vestibular-intact mice will immediately right themselves in both the tests (18, 24).

#### Open Field Test

**Figure 2:**
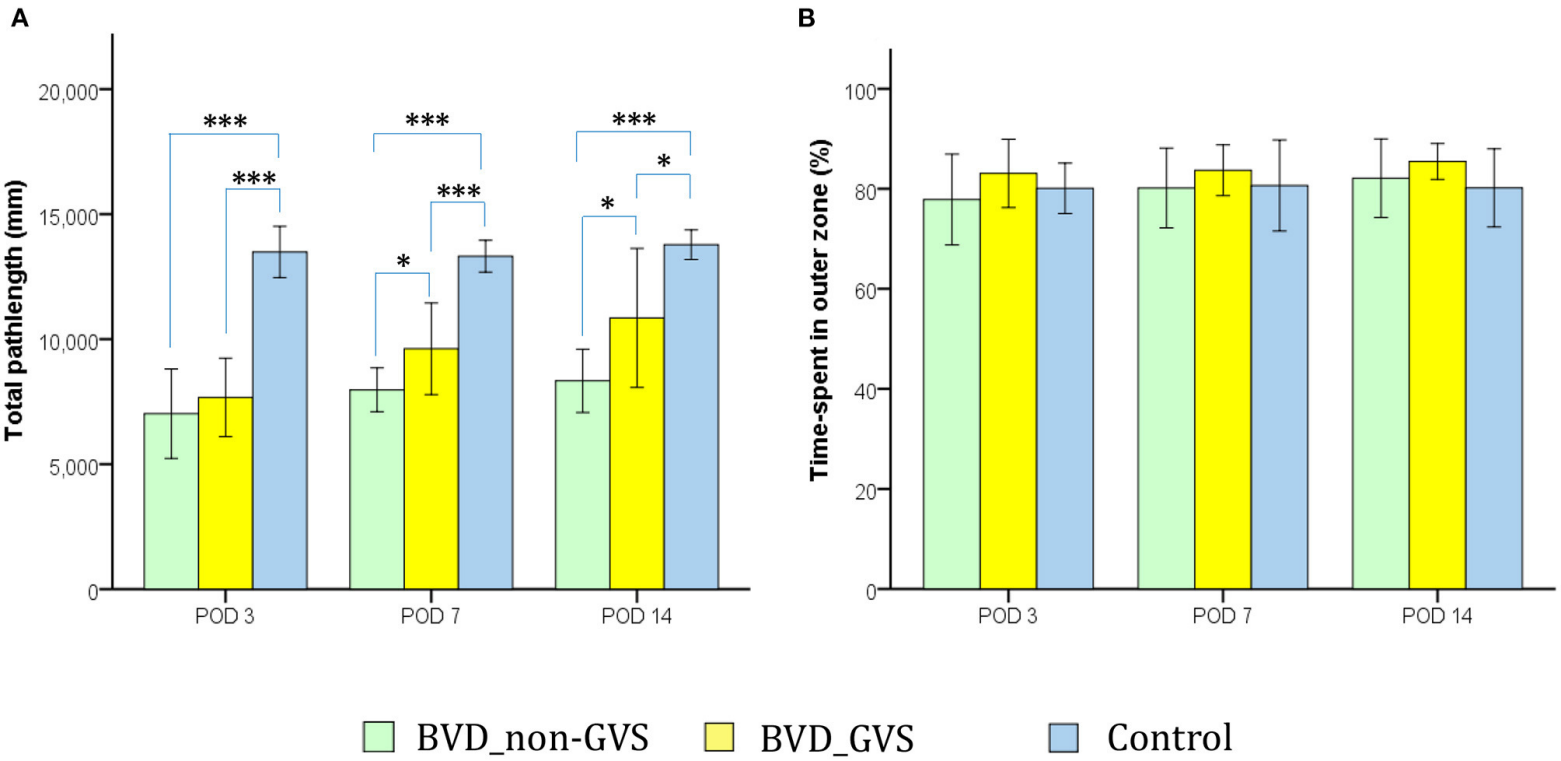
Evaluation of the locomotor activity of mice in an open-field (OF) task. Locomotion impairment was indicated by a significant decrease in the total path length. The values of the BVD_non-GVS group were lower than those of the control group on PODs 3 (*p* < 0.001, Bonferroni test), 7 (*p* < 0.001, Bonferroni test), and 14 (*p* < 0.001, Bonferroni test). GVS improved the total path length during OF activity on PODs 7 (*p* = 0.031, LSD test) and 14 (*p* = 0.027, LSD test) **(A)**. The percentage of time spent in the outer zone, which is an indicator of anxiety, did not differ among the three groups **(B)**. Values are indicated as the mean ± SD. Statistical significances were calculated using one-way ANOVA with post-hoc tests. *Significant differences between two groups: * *p* < 0.05; *** *p* < 0.001.

The OF apparatus and the protocols for assessing the locomotor activities of the mice have been documented in our previous papers (14, 15, 30). In which, we employed a circular arena (37 cm diameter × 53 cm height) and an overhead tracking camera (HD 1080p C920, Logitech, Switzerland) mounted at the center. With the 2-min task for mice, we sought to collect 2 major metrics comprising the total path length across the whole ground (mm) (Figure 2A), and the percentage of time spent in the outer (peripheral) zone as an indicator of anxiety (31) (Figure 2B).

#### Y Maze

**Figure 3:**
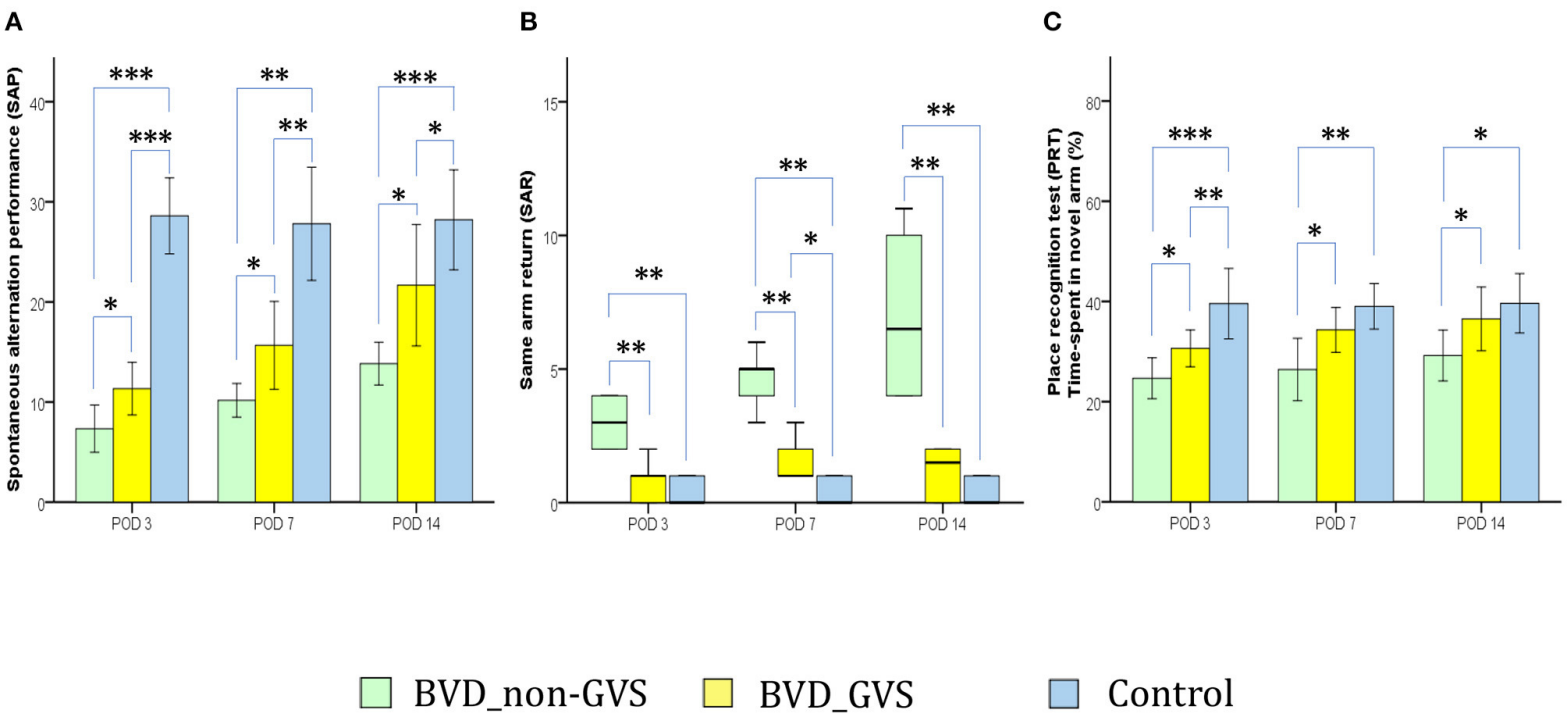
Evaluation of locomotor activity and spatial navigation in the Y maze test. The spontaneous alternation performance (SAP), an indicator of spatial working memory and locomotor activity, was decreased in the BVD_non-GVS group compared with the control group on PODs 3 (*p* < 0.001, Bonferroni test), 7 (*p* < 0.001, Tamhane test), and 14 (*p* < 0.001, Bonferroni test). The values of the BVD_GVS group were better than those of the BVD_non-GVS group on PODs 3 (*p* = 0.018, LSD test), 7 (*p* = 0.049, Tamhane test), and 14 (*p* = 0.02, Bonferroni test) **(A)**. The same arm return (SAR), which indicates the degree of attentional difficulty during active working-memory performance, was significantly higher in the BVD_non-GVS group than in the control group on PODs 3 (*p* = 0.005, Mann-Whitney U test), 7 (*p* = 0.005, Mann-Whitney U test), and 14 (*p* = 0.005, Mann-Whitney U test). The BVD_GVS group showed better values than the BVD_non-GVS group on PODs 3 (*p* = 0.006, Mann-Whitney U test), 7 (*p* = 0.004, Mann-Whitney U test), and 14 (*p* = 0.003, Mann-Whitney U test) **(B)**. In the PRT, an indicator of spatial reference memory, the BVD_non-GVS group scored significantly worse than the control mice on PODs 3 (*p* < 0.001, Bonferroni test), 7 (*p* = 0.002, Bonferroni test), and 14 (*p* = 0.017, Bonferroni test). The PRT performance improved significantly in the BVD_GVS group, compared with the BVD_non-GVS group, on PODs 3 (*p* = 0.033, LSD test), 7 (*p* = 0.038, Bonferroni test), and 14 (*p* = 0.031, LSD test) **(C)**. The SAP and PRT values are given as the mean ± SD, and the *p* values were calculated using one-way ANOVA with *post-hoc* tests. The SAR values are given as the median (95% confidence interval), and those *p* values were calculated using the Kruskal-Wallis test and Mann-Whitney U-test. *Significant differences between two groups: * *p* < 0.05; ** *p* < 0.01; *** *p* < 0.001.

The Y maze apparatus and the protocols for measuring the short-term memory have been described in our previous papers (14, 15). In which, a plastic Y-shaped maze with three arms (51 × 18 × 32 cm; labeled A, B, C) orientated at 120 angles from each other and an overhead tracking camera (HD 1080p C920, Logitech, Switzerland) mounted at the center were employed. With the 6-min task for mice, we sought to gather three principal parameters that reflect short-term memories comprising; (i) the spontaneous alternation performance (SAP: defined as entries into all the three arms consecutively, e.g. ABC, BCA…) represents the spatial working memory (32, 33) (Figure 3A), (ii) the same arm return (SAR: defined as visiting the same arm repeatedly, e.g., AA, BB, CC) represents the spatial working memory error (33, 34) (Figure 3B), and (iii) the place recognition test [PRT: defined as the percentage of time spent in the B arm (after unblocking)—designated as the novel arm] represents the spatial working and reference memory (14, 15, 33) (Figure 3C). We measured these SAP, SAR, and PRT scores at baseline and PODs 3, 7, and 14 (Figure 1).

#### Morris Water Maze Task

**Figure 4:**
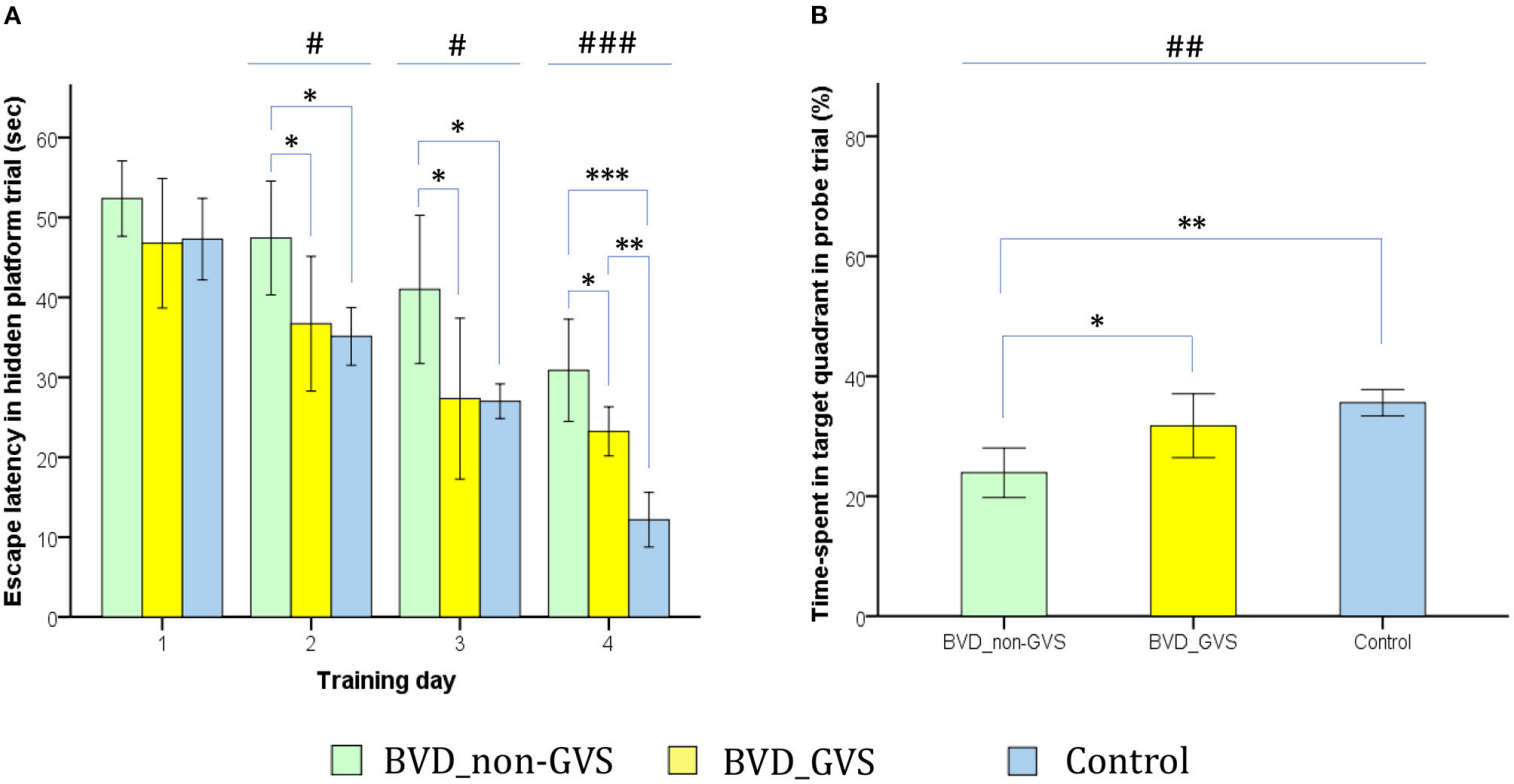
Evaluation of long-term spatial reference memory using the Morris water maze (MWM). The escape latencies to find the hidden platform gradually decreased through the training sessions, indicating ongoing learning. Longer values of escape latency to find the hidden platform indicate an inadequate acquisition of spatial memory and navigation. Differences between the groups were observed on training days (TDs) 2 (*p* = *0*.012, ANOVA), 3 (*p* = *0*.013, ANOVA), and 4 (*p* < 0.001, ANOVA). The BVD_non-GVS group had longer escape latency than the control group on TDs 2 (*p* = 0.022, Bonferroni test), 3 (*p* = 0.032, Bonferroni test), and 4 (*p* < 0.001, Bonferroni test). The BVD_GVS group had shorter escape latency than the BVD_non-GVS group on TDs 2 (*p* = 0.037, Bonferroni test), 3 (*p* = 0.028, Bonferroni test), and 4 (*p* = 0.024, Bonferroni test) **(A)**. Residual impairments in long-term spatial memory were also indicated by a lower percentage of time spent in the target quadrant (probe trial) on POD 14 in the BVD_non-GVS compared with both the control (*p* = 0.001, Bonferroni test) and BVD_GVS groups (*p* = 0.012, Bonferroni test) (*p* = 0.001, ANOVA) **(B)**. The values are indicated as the mean ± SD. Statistical significance was calculated using one-way ANOVA with post-hoc tests. *Significant differences between two groups; #significant differences among three groups: *, ^#^*p* < 0.05; **, ^##^*p* < 0.01; ***, ^###^*p* < 0.001.

The MWM was used to evaluate spatial memory and navigation, and the apparatus and experimental protocols as described in our earlier works (14, 15). The paradigm was comprised of a plastic circular water tank (175 diameter and 62 cm high) with opaque made water, an acrylic circular escape platform (15 cm in diameter), four distal visual cues corresponding to four directions (east, south, west, and north), and a camera (HD 1080p C920; Logitech International SA, Lausanne, Switzerland) mounted in the center above the tank. Given the 24-h intervals between the last training session and the probe trial session, MWM was appropriate for assessing the long-term memory and learning process (20, 35–37). We sought to collect two major parameters, (i) the amount of time that elapsed before the animal climbed onto the platform to escape the water (escape latency) during the hidden platform training session (Figure 4A) (ii) the percentage of time spent in the target quadrant (SE quadrant) during the probe trial (Figure 4B). We also examined an auxiliary metric, the mean swim velocity using a visible platform test done 30 min following the probe trial to assess sensorimotor ability and motivation (38).

### Statistical Analysis

We analyzed the data with SPSS Statistics version 23.0 (IBM Corp., Armonk, NY, USA). The normality of the distribution was assessed using the Shapiro-Wilk test for each parameter. The repeated measures ANOVA or the Friedman Tests were used to analyze the interaction between surgical conditions—time as a first-level analysis. The parametric variables are shown as the mean ± SD, and statistical significances were calculated using *post-hoc* one-way ANOVA accompanied by a test of the homogeneity of variances (Levene test): (i) if *p* > 0.05, ANOVA (between-group comparison) and the LSD test or Bonferroni test (multiple comparisons) were used; (ii) if *p* < 0.05, the Robust test (between group comparison) and Tamhane test (multiple comparisons) were used. Non-parametric variables are given as the median [interquartile range], and significant differences were determined using the Kruskal-Wallis test (between-group comparison) accompanied by the Mann-Whitney *U* test or the Wilcoxon signed-rank test (pairwise comparisons). All the tests were performed at a 0.05 level of significance.

## Results

The results of the air-righting and contact-righting tests confirmed the vestibular dysfunction in all the BL mice; the control mice-righted themselves instantly. In the air-righting reflex test examining the ability to right themselves in the air, the control mice all landed on their feet, whereas the BL mice tended to land on their backs or sides. In the contact-righting test examining their behavior when placed supine on a horizontal surface, the control mice-righted themselves, whereas the BL mice lay supine and did not right themselves. The BL mice were observed moving in circles, swayed their head, and curled up when pulled up by the tail. These vestibular symptoms have severely occurred the first 2 days postsurgery; after that, BL mice could stand unaided on a tilt platform and walk. Considering this natural recovery course, we conducted all the subsequent behavioral investigations beginning on POD 3, when all mice were free from the limitations of motor coordination problems (Figure 1).

### Galvanic Vestibular Stimulation Effect on Locomotion in BL Mice

Bilateral labyrinthectomy caused a locomotion impairment that was indicated by a significant decrease in the total path length in the OF test. The values of the BVD_non-GVS group were lower than those of the control group on PODs 3 (*p* < 0.001, Bonferroni test), 7 (*p* < 0.001, Bonferroni test), and 14 (*p* < 0.001, Bonferroni test). GVS improved the total path length during OF activity on PODs 7 (*p* = 0.031, LSD test) and 14 (*p* = 0.027, LSD test) (Figure 2A).

We also used the OF task to quantify the effects of anxiety on this experimental findings by using an indicator, the percentage of time spent in the outer zone (31, 39, 40). Because of there being no significant differences among the groups in this metric, we could likely exclude the effects of anxiety on locomotor and spatial cognition results in this study (Figure 2B).

### Spatial Cognition in BL Mice and GVS Effects

The alterations of spontaneous alternation performance (SAP) and same arm return (SAR) scores of the Y maze test have partially demonstrated the deficits of the short-term visuospatial cognitive alternation performance following BL. For instance, playing as an indicator of both the locomotor activity and spatial working memory, SAP was lower in the BVD_non-GVS group than in the control group on PODs 3 (*p* < 0.001, Bonferroni test), 7 (*p* < 0.001, Tamhane test), and 14 (*p* < 0.001, Bonferroni test). The values of the BVD_GVS group were better than that of the BVD_non-GVS group on PODs 3 (*p* = 0.018, LSD test), 7 (*p* = 0.049, Tamhane test), and 14 (*p* = 0.02, Bonferroni test) (Figure 3A). Meanwhile, the values of SARs, reflected the degree of attentional difficulty during an active working-memory task, were significantly higher in the BVD_non-GVS group than in the control group on PODs 3 [3 (2.06–3.94) turns, Z = −2.79, *p* = 0.005, Mann-Whitney *U* test], 7 [5 (3.58–5.75) turns, Z = −2.796, *p* = 0.005, Mann-Whitney *U* test], and 14 [6.5 (3.49–10.51) turns, Z = −2.798, *p* = 0.005, Mann-Whitney *U* test]. The BVD_GVS group showed better values than the BVD_non-GVS group on PODs 3 (Z = −2.776, *p* = 0.006, Mann-Whitney *U* test), 7 (Z = −2.879, *p* = 0.004, Mann-Whitney *U* test), and 14 (Z = −2.929, *p* = 0.003, Mann-Whitney *U* test) (Figure 3B).

Serving as an indicator of spatial reference memory, the PRT was significantly lower in the BVD_non-GVS group than in the control mice on PODs 3 (24.66 ± 3.91% vs. 39.56 ± 5.66%, *p* < 0.001, Bonferroni test), 7 (26.41 ± 5.93% vs. 39.03 ± 3.66%, *p* = 0.002, Bonferroni test), and 14 (29.19 ± 4.84% vs. 39.6 ± 4.78%, *p* = 0.017, Bonferroni test). The mean time spent in the newly unblocked arm increased significantly in the BVD_GVS group compared with the BVD_non-GVS group on PODs 3 (*p* = 0.033, LSD test), 7 (*p* = 0.038, Bonferroni test), and 14 (*p* = 0.031, LSD test) (Figure 3C).

Morris water maze task was sought to assess long-term visuospatial memory and learning process (20, 35–37). Accordingly, the ongoing learning was indicated by the progressive decrease across the training sessions of the escape latencies to find the hidden platform. Longer escape latency values in finding the hidden platform reflected an impairment of spatial memory and navigation. We noticed the differences among the groups on training days (TDs) 2 (*p* = *0*.012, ANOVA), 3 (*p* = *0*.013, ANOVA), and 4 (*p* < 0.001, ANOVA). The BVD_non-GVS group had higher escape latency values than the control group on TDs 2 (47.43 ± 6.79 s vs. 35.12 ± 2.9 s, *p* = 0.022, Bonferroni test), 3 (41 ± 8.84 s vs. 27 ± 1.75 s, *p* = 0.032, Bonferroni test), and 4 (30.87 ± 6.09 s vs. 12.17 ± 2.76 s, *p* < 0.001, Bonferroni test). The escape latency was shortened in the BVD_GVS group compared with the BVD_non-GVS group on TDs 2 (*p* = 0.037, Bonferroni test), 3 (*p* = 0.028, Bonferroni test), and 4 (*p* = 0.024, Bonferroni test) (Figure 4A).

The residual impairments in long-term spatial memory exhibited in the MWM task were also indicated by a worse probe trial performance on POD 14, that is a lower percentage of time spent in the target quadrant in the BVD_non-GVS group (23.9 ± 3.93%) compared with the control (35.57 ± 1.76%, *p* = 0.001, Bonferroni test) and BVD_GVS groups (31.73 ± 5.08%, *p* = 0.012, Bonferroni test) (*p* = 0.001, ANOVA) (Figure 4B). Meanwhile, the BVD_GVS group displayed a negligible difference from the control group (*p* = 0.131, LSD test). The differences in the MWM learning activities were not derived from motor deficits (41, 42) because the groups did not differ significantly in mean swim velocity (*p* > 0.05, LSD test).

## Discussion

Our data demonstrated the effects of the early GVS intervention on the short-term and long-term spatial memory, navigation, and locomotion deficits induced by acute BVD in a mouse model.

### Locomotion and Spatial Cognition Impairments in BVD

Given its crucial role in integrating and converging multisensory information between the ipsi- and contralateral sides of multilevel brain regions, the relationship between bilateral impairment of vestibular system (BVD) and deficits of visuospatial cognitive performance has fascinated scientific interests for the past decades (17–19). Our experimental-based current findings could add an evidence that the BVD induces dysfunctions in spatial memory and navigation from the acute phase to at least 2 weeks after BL (17–19). Other neurophysiological investigations have suggested multiple pathways in which the vestibular signals project to the hippocampus along with other medial temporal lobe regions, to build up maps of 3D space for the development of spatial memory during learning tasks (43, 44).

In the Y maze, the SAP is driven by the innate curiosity and exploratory behavior of mice for the novel surroundings and requires good spatial working memory to recognize which arms have recently been visited (45). During the PRT, mice need to memorize the relationship between distal spatial cues and the arm that had previously been blocked to recognize it as novel, and thus, visit it more frequently than the other arms (33). Both the SAP and PRT have previously been used to measure spatial working and reference memory (32), especially short-term memory. The MWM is also designed to assess hippocampal-dependent spatial navigation and reference memory, especially in the place learning, and extensive evidence of its validity is available (20, 41, 46). Compared to the Y maze, the MWM is more specialized for hippocampal-dependent spatial navigation than the PRT since it eliminates the use of non-spatial or proximal cues, such as odor trail interference, to solve the maze (46). Furthermore, the 24-h interval between the training session and the probe trial session allows the MWM to reflect long-term memory or the consolidation process in the hippocampus rather than the immediate and short-term effects of bilateral vestibular loss (20, 35–37). It has been shown that short-term spatial memory is not sequentially linked with the different stages of long-term spatial memory, even though there was a time-dependent consolidation of the newly established memory into the long-term memory (35, 47).

In line with our findings, deficiencies in gait, locomotor activity, and spatial memory following BVD have been extensively described in both humans and other animals (17, 48–51). Vestibular signals make major contributions to balance and spatial memory functions *via* multisensory convergent and multimodal signaling pathways (1, 2, 11, 12). Concerned to the physiological formation of spatial memory and navigation, there were two major underlying components: (i) a continuous representation of the location and motion of the individual whose coordinates are provided mainly by vestibular and visual cues, and (ii) spatial memory processing in the hippocampal formation (20). As a result, inhibiting the vestibular signals in the pathways to the hippocampus, such as peripheral vestibular lesions, likely led to disrupt the function of hippocampal cells *in vivo* and hippocampal field potentials *in vitro* and cause long-term changes in hippocampal neurochemistry (18). Patients with chronic BVD experience bilateral hippocampal atrophy (20), particularly a reduction in hippocampal gray matter volume in the Cornu ammonis 3, which plays an important role in the formation of episodic memory and the acquisition of spatial information within short-term memory in tasks that require rapid encoding, novelty detection, working memory, and recall of primarily spatial information (21). Hippocampal atrophy predominantly impaired complex forms of visuospatial memory processing, even as non-spatial functions, which additionally rely on the surrounding medial temporal lobe and prefrontal tissue, remain well preserved (20). Morphometric changes in BVD subjects have also been shown to be time-dependent and take place in parallel with adaptive mechanisms (21). Alternatively, in some recent behavioral studies, BL was found to disrupt spatial learning, primarily in the absence of visual signals, because of oscillopsia (52), which is thought to be the result of difficulties in localization caused by problems with self-movement monitoring (53), a function ascribed to the hippocampus.

### Galvanic Vestibular Stimulation Effects on Locomotion and Spatial Cognition in BVD Mouse Model

Intriguingly, this work has shown that a short-term GVS intervention conferred benefits on balance control, locomotion, and short- and long-term spatial memory following acute BL, which resulted in better behavioral outcomes more than a week after the cessation of the intervention. Our findings are consistent with previous studies, which also revealed that GVS had positive effects on postural control and locomotion in BVD (28, 29, 54–56). Meanwhile, one study revealed that 6-sessions of GVS induced changes in tactile extinction that lasted up to 1-year follow-up (57).

In this study, the significant increases we found in the total path length during the OF test and SAP of the Y maze task after the GVS intervention imply that the GVS positively affected the recovery of locomotor and postural control deficits following BL. The GVS affected the vestibulo-spinal and other non-dopaminergic pathways probably through a neuromodulation mechanism of facilitating effect on the vestibular nuclei (VN). Neuronal sensitivity to GVS increases with discharge variability, whereby the thick, fast-conducting irregularly firing afferents are more sensitive than the thin, slower-conducting regularly firing vestibular afferents for both the cathode and anode (26, 58–60). Contrast to the slower-conducting regularly firing afferents are prevalent in the input of the VOR pathway, the fast-conducting irregularly firing afferent predominates in the VSR pathway (26, 61) that underlie postural dysfunction after BL (62–64). Therefore, the GVS intervention can restore locomotor function by modulating type I hair cells (58, 60), which show an irregular phasic signal (65, 66), and can accelerate vestibulo-motor compensation during the acute period after BL.

The beneficial effects of GVS on the recovery of visuospatial memory deficits following BL could be explained with the implications of the reestablishment of four proposed vestibular-hippocampal signaling pathways: vestibulo-thalamo-cortical, vestibulo-cerebello-cortical, head direction, and theta pathways (5, 6). Previous studies revealed that the electrical stimulation excited the medial VN and increased the firing rates of hippocampal CA1 complex spike cells, which correspond to place cells (67). Similarly, GVS at the ampulla of the SCCs generated the initiation of theta activity in the numerous areas of the hippocampal formation (44), and it can be speculated that GVS improves neuronal activity for spatial orientation (68). It has been also known that the critical involvement of the hippocampal theta rhythm in visuospatial information processing and regulating self-movement signals (68, 69). Furthermore, GVS enhances the neuronal activation demonstrated *via* an increase in c-Fos positive cells in the hippocampus following the multiple-session administration (29, 70). Besides, based on the concept of sensory substitution, strengthening the function of other sensory systems, such as visual (30, 71) and somatosensory functions (72) and boosting reciprocal intersensory interactions could be considered a beneficial influence of the GVS to enhance efficiency in the spatial memory and navigation activities (73). All the aforementioned mechanisms just assist us somewhat in imagining the explanation of the GVS effects on strengthening the function of spatial navigation. To acquire a comprehensive understanding, further in-depth investigations are required.

Although GVS has been shown to be effective in ameliorating both the motor impairments and short- and long-term spatial memory deficits induced by BVD that recovery did not appear to approach the baseline level. Expanding the number of GVS sessions to more than five might improve the effectiveness. A second strategy to maximize the effect of GVS could be the use of different modes of stimulation, such as subthreshold noisy GVS, which is based on the principle of stochastic resonance, in which a subthreshold sensory input can be upgraded to exceed a specified threshold when being augmented by a considerably higher-frequency noise signal (74). Therefore, further investigations should be conducted to find the optimal stimulating parameter for GVS in each disease model.

In conclusion, our data demonstrate that the early administration of sinusoidal GVS accelerated the recovery of both locomotion and spatial memory and navigation deficiencies, although the efficacy remained limited. Nonetheless, our promising early results allow us to hope that GVS can be used to treat patients with bilateral vestibulopathies who suffer from motor coordination and spatial cognitive difficulties.

## Data Availability Statement

The original contributions presented in the study are included in the article/supplementary material, further inquiries can be directed to the corresponding author/s.

## Ethics Statement

The animal study was reviewed and approved by the Animal Care Committee of the Gachon University of Medicine and Science (IRB MRI2019-0008).

## Author Contributions

TTN: writing the article, analyzing the data and making the figures. G-SN: analyzing the data and conducting the experiment. GCH: analyzing the data and conducting the experiment. CL: analyzing the data. S-YO: conceptualizing the study and interpretation the data and writing the article.

## Funding

This work was supported by a National Research Foundation of Korea (NRF) Grant funded by the Korean Government (Ministry of Science and ICT) (No. 2022R1A2B5B01001933) and by the Fund of Biomedical Research Institute, Jeonbuk National University Hospital.

## Conflict of Interest

The authors declare that the research was conducted in the absence of any commercial or financial relationships that could be construed as a potential conflict of interest.

## Publisher's Note

All claims expressed in this article are solely those of the authors and do not necessarily represent those of their affiliated organizations, or those of the publisher, the editors and the reviewers. Any product that may be evaluated in this article, or claim that may be made by its manufacturer, is not guaranteed or endorsed by the publisher.
